# RG-PSR: Reliability-Guided Poisson Surface Reconstruction for Degraded 3D-Imaging Point Clouds

**DOI:** 10.3390/jimaging12080369

**Published:** 2026-08-10

**Authors:** Na Liu, Fan Zhang, Jiawei Wang, Dan Zhang, Jinliang Wu, Xiaohui Li

**Affiliations:** 1 Information Science Academy of China Electronics Technology Group Corporation, Beijing 100043, China; 201831210007@mail.bnu.edu.cn (N.L.); fzhang@mail.bnu.edu.cn (F.Z.); wujinliang@cetcisa.com (J.W.); lixiaohui@cetcisa.com (X.L.); 2 School of Computer Science, Qinghai Normal University, Xining 810008, China; wjwaid1314@163.com; 3 State Key Laboratory of Tibetan Intelligence, Xining 810008, China; 4 Key Laboratory of Tibetan Information Processing, Ministry of Education, Xining 810008, China

**Keywords:** 3D point cloud processing, surface reconstruction, Poisson reconstruction, mesh quality, reliability estimation, normal refinement, adaptive trimming, kNN filtering

## Abstract

Three-dimensional (3D) imaging systems, including depth cameras, LiDAR sensors, and multi-view scanning pipelines, often produce point clouds with noisy normals, outliers, sparse sampling, and non-uniform density, which can degrade downstream mesh reconstruction. Poisson surface reconstruction is lightweight and training-free, but its global implicit formulation is sensitive to unreliably oriented samples and fixed density-trimming thresholds. This paper presents RG-PSR, a reliability-guided enhancement framework for Poisson-family surface reconstruction from degraded 3D-imaging point clouds. RG-PSR estimates a deterministic per-point reliability score from local density regularity, spacing variation, and normal consistency, and propagates this score through conservative point filtering, reliability-guided normal refinement, adaptive density-reliability trimming, and structure-aware postprocessing. The main pipeline requires no manual labels, neural network training, or ground-truth meshes at inference time. Experiments on three groups of object meshes under five deterministic degradation types show that RG-PSR improves Poisson-family reconstruction under degraded inputs. Compared with fixed density-trimmed Poisson reconstruction, RG-PSR reduces the overall Chamfer-$L_{1}$ from 0.0218 to 0.0172, improves $F_{0.01}$ from 0.6618 to 0.6836, and reduces ${\mathrm{Artifact}}_{0.02}$ from 0.3090 to 0.2632. In the broader classical comparison, local triangulation methods achieve stronger point-wise accuracy, while RG-PSR yields the fewest connected components and the highest largest-component ratio. These results position RG-PSR as a practical reliability layer for coherent Poisson-family reconstruction rather than a universal replacement for all surface-reconstruction methods.

## 1. Introduction

Three-dimensional imaging systems, including structured-light scanners, depth cameras, LiDAR sensors, range-imaging devices, and multi-view stereo pipelines, increasingly produce dense point clouds as geometric observations of physical objects and scenes. To support downstream applications such as visualization, measurement, simulation, manufacturing, robotic perception, digital heritage, and geometric analysis, these point clouds are routinely converted into triangular meshes or implicit surfaces. Surface reconstruction from point clouds, therefore, occupies a central position in 3D imaging, computer vision, computer graphics, and geometry processing [1,2,3,4].

Despite substantial progress, robust reconstruction from practical 3D imaging data remains an open challenge. Real scanned point clouds are rarely clean, uniformly sampled, or correctly oriented. They commonly contain noisy surface samples, inaccurate or unoriented normals, statistical outliers, missing regions, sparsely covered structures, non-uniform density, anisotropic sampling, and mixed degradation patterns. These imperfections compromise not only point-wise surface accuracy but also mesh usability: a reconstructed mesh may achieve acceptable geometric distance to the ground truth while still containing open boundaries, disconnected components, floating sheets, non-manifold edges, or weakly supported fragments. A practical reconstruction framework must, therefore, balance both geometric fidelity and structural coherence.

Classical surface-reconstruction methods remain widely deployed because they are interpretable, lightweight, and free of supervised training data. Local triangulation methods such as alpha shapes, the Ball Pivoting Algorithm (BPA), and Greedy Projection Triangulation (GP3) can faithfully preserve observed samples when the input point cloud is dense, clean, and reasonably uniform [5,6,7,8]. However, these methods are sensitive to neighborhood scale, sampling density, local connectivity, and normal quality. Under sparse, noisy, or strongly non-uniform sampling, they frequently produce fragmented surfaces, open boundaries, or unstable mesh connectivity.

Poisson surface reconstruction offers an alternative implicit formulation: rather than triangulating local neighborhoods directly, it solves for an indicator function whose gradient field is consistent with the input normal field and then extracts an isosurface from the result [9]. Screened Poisson reconstruction further improves data fidelity by adding point-interpolation constraints while preserving the global formulation [10]. These Poisson-family methods are attractive in 3D-imaging pipelines because they are training-free, globally regularized, and capable of producing smooth, coherent closed meshes. Their principal limitation, however, is a strong dependence on the reliability of the oriented input samples: noisy normals distort the reconstructed vector field, outliers introduce weakly supported sheets, sparse regions are over-smoothed, and non-uniform density causes unstable trimming behavior. Normal estimation and orientation are themselves non-trivial under noise, sharp features, incomplete observations, and degraded sampling [11,12,13,14].

Learning-based reconstruction methods have produced competitive results by learning implicit fields, local shape priors, or differentiable reconstruction layers from training data [15,16,17,18,19]. These results demonstrate the value of learned priors for reconstructing detailed surfaces. They typically require task-specific training data, network design effort, and additional computational resources, and their performance may degrade under distribution shifts between training shapes and degraded 3D-imaging inputs. In many practical deployment scenarios, particularly when labeled training data are unavailable or when new sensor geometries are introduced, deterministic, training-free reconstruction pipelines retain significant practical value.

This work focuses on improving Poisson-family reconstruction under degraded 3D-imaging point-cloud inputs. Rather than proposing a universal replacement for all surface-reconstruction methods, we address a more targeted question: can a lightweight, deterministic reliability mechanism make Poisson reconstruction substantially more robust when dealing with unreliable samples, unstable normals, and weakly supported mesh regions? To answer this question, we propose RG-PSR (Reliability-Guided Poisson Surface Reconstruction). The core idea is to estimate a per-point reliability score from local geometric evidence and to propagate this score throughout the reconstruction pipeline. Specifically, RG-PSR combines local density regularity, spacing variation, and normal consistency into a single reliability score, which is then used for conservative point filtering, reliability-guided normal refinement, and adaptive density-reliability trimming after Poisson reconstruction.

We evaluate RG-PSR on normalized object meshes under representative degradation types, including noise, sparsity, non-uniform density, outliers, and mixed degradation. The results show that RG-PSR consistently improves Poisson-family reconstruction over vanilla Poisson and fixed density trimming, especially in terms of artifact suppression and mesh coherence. Local triangulation methods such as BPA and GP3 can still achieve lower point-wise error on dense and favorable inputs; therefore, RG-PSR is positioned as a lightweight reliability layer for Poisson-family reconstruction rather than a universal replacement for all classical surface-reconstruction methods.

The main contributions of this work are as follows:

- A deterministic per-point reliability estimation strategy for degraded 3D-imaging point clouds, which combines local density regularity, spacing variation, and normal consistency into a unified reliability score.
- A training-free reliability-guided Poisson reconstruction pipeline that propagates point reliability through conservative filtering, normal refinement, adaptive density-reliability trimming, and structure-aware mesh postprocessing.
- An evaluation protocol and analysis that separate point-wise geometric accuracy from mesh structural quality, clarifying the trade-off between local triangulation accuracy and Poisson-family mesh coherence under degraded inputs.

The remainder of this paper is organized as follows. Section 2 reviews classical geometric reconstruction, Poisson-family methods, robust normal and reliability estimation, and learning-based reconstruction approaches. Section 3 presents the proposed RG-PSR framework, including reliability estimation, reliability-guided preprocessing, Poisson reconstruction, adaptive support trimming, and structure-aware mesh repair. Section 4 presents the experimental setup, comparisons, sensitivity and ablation studies, reliability validation, a LiDAR/scanning case study, and discussion. Section 5 concludes the paper and outlines future work.

## 2. Related Work

This section reviews the literature most relevant to the proposed RG-PSR framework. We first summarize surface reconstruction from 3D imaging point clouds and classical geometric reconstruction methods. We then discuss implicit and Poisson-family reconstruction, which forms the reconstruction backbone of our method. Next, we review robust normal estimation, reliability analysis, and reconstruction under degraded inputs. Finally, we briefly discuss learning-based and hybrid surface-reconstruction methods. In contrast to methods that introduce a new implicit representation or rely on learned priors, RG-PSR focuses on a lightweight reliability-guided enhancement of the classical Poisson-family pipeline.

### 2.1. Surface Reconstruction from 3D-Imaging Point Clouds

Surface reconstruction from point clouds is a long-standing problem at the intersection of 3D imaging, computer vision, computer graphics, and geometry processing. Early range-image fusion methods demonstrated that scanned depth observations can be integrated into volumetric representations for building complex 3D models [1]. Since then, surface reconstruction has been extensively studied under a wide range of assumptions about sampling quality, noise characteristics, surface topology, and output representation [2,3,4]. Practical 3D-imaging data, however, routinely contain sensor noise, missing observations, outliers, non-uniform density, and unstable normals, making reconstruction more difficult than idealized clean-and-uniform settings.

This work operates in the practical degraded-input setting. Rather than assuming perfect oriented samples, it examines how a lightweight reliability mechanism can improve the robustness of a widely used, training-free Poisson-family reconstruction pipeline.

### 2.2. Classical Geometric and Local Triangulation Methods

Classical surface-reconstruction methods remain attractive for their interpretability, computational efficiency, and independence from supervised training data. Hoppe et al. introduced a foundational approach in which a signed distance function is estimated from unorganized points and a polygonal surface is subsequently extracted [20]. Alpha shapes infer the shape of a point set through a scale-controlled simplicial complex [5,6]. Voronoi- and Delaunay-based methods, including the Crust, Power Crust, and Cocone family, established theoretical foundations for reconstructing surfaces from sufficiently dense, noise-free samples [21,22,23].

Local triangulation methods construct triangles directly from nearby samples. BPA reconstructs an interpolating triangular mesh by rolling a virtual ball over the point cloud [7], while GP3 grows local surface patches by projecting neighborhoods onto estimated tangent planes [8]. These methods achieve strong point-wise geometric accuracy when the input is dense and locally uniform, but their dependence on neighborhood scale, sampling density, and normal quality can lead to fragmented components, open boundaries, or unstable topology under sparse, noisy, or non-uniform sampling conditions. This behavior motivates complementary approaches that prioritize global mesh coherence and artifact suppression.

### 2.3. Implicit and Poisson-Family Surface Reconstruction

Implicit reconstruction methods represent surfaces as level sets of scalar fields. Radial basis function reconstruction [24], point set surfaces and algebraic variants [25,26,27], robust moving least squares [28], point-cloud consolidation [29], and smooth signed distance formulations [30] have shown that implicit representations improve surface smoothness and continuity, though they may require careful parameter selection under non-uniform sampling.

Poisson surface-reconstruction formulates the problem as a global least-squares solve for an indicator function consistent with the input normal field, followed by isosurface extraction [9]. Screened Poisson reconstruction incorporates point-interpolation constraints to improve data fidelity [10]. These Poisson-family methods are training-free, globally regularized, and routinely produce smooth, closed meshes. Their principal sensitivity lies in the quality of the oriented input samples: noisy normals distort the recovered vector field, outliers create unsupported sheets, sparse regions are over-smoothed, and non-uniform density destabilizes the density-trimming stage.

Iterative Poisson surface reconstruction (iPSR) alternates Poisson reconstruction with normal re-orientation, enabling reconstruction from unoriented points [31]. It is complementary to RG-PSR: iPSR targets global orientation through repeated Poisson solves, whereas RG-PSR retains a single solve and uses per-point reliability to guide filtering, normal refinement, and support trimming. Combining the two is left for future work. RG-PSR is positioned within this Poisson-family line of work; it does not introduce a new implicit representation but instead augments the classical Poisson pipeline with per-point reliability estimation, reliability-guided normal refinement, and adaptive density-reliability trimming.

### 2.4. Robust Reconstruction, Normal Reliability, and Degraded Inputs

Robust reconstruction from degraded point clouds requires reasoning beyond simple geometric proximity. In practical 3D-imaging data, a low-density region may correspond to an outlier or to a valid sparse structure; similarly, a low-density reconstructed region may be an artifact or a meaningful thin surface. Decisions based on a single geometric cue are, therefore, unreliable under mixed degradation.

Normal estimation and orientation are especially critical for Poisson-family methods. Classical local PCA normals perform well on smooth, dense point clouds but become unstable near sharp features, noise, outliers, missing regions, and non-uniform sampling. Learning-based approaches such as PCPNet [12] improve local shape property estimation from raw point clouds, while other methods pursue globally consistent normal orientation via deep networks, dipole propagation, or winding-number optimization [11,13,14,32]. These results highlight normal reliability as a central bottleneck for downstream surface reconstruction.

RG-PSR addresses degraded inputs by combining multiple local geometric cues (density regularity, spacing variation, and normal consistency) into a single per-point reliability score, which is then propagated into normal refinement and mesh support estimation rather than used solely for point removal. The evaluation additionally reports structural mesh metrics (boundary edges, boundary loops, connected components, largest-component ratio, artifact ratio, and missing ratio) alongside point-wise geometric metrics, because a method may achieve low Chamfer distance while producing fragmented or structurally unusable meshes.

### 2.5. Learning-Based and Hybrid Surface Reconstruction

Learning-based surface-reconstruction methods have recently achieved strong performance on clean benchmark datasets. Neural implicit methods such as DeepSDF and Occupancy Networks learn continuous signed-distance or occupancy fields over large shape collections [15,16]. Convolutional Occupancy Networks improve scalability by combining convolutional encoders with implicit occupancy decoders [33]. Sign-agnostic learning and implicit geometric regularization reduce the reliance on fully signed supervision [34,35], and Neural-Pull learns signed distance functions by iteratively pulling query locations toward the nearest surface point [36].

Methods more directly targeting point-cloud surface reconstruction include Points2Surf [17], which learns implicit surfaces from patch-level features without requiring oriented normals, and POCO [19], which attaches learned features to input points using point convolutions. Shape As Points introduces a differentiable Poisson solver, bridging oriented point representations and mesh extraction within a differentiable learning framework [18].

These learning-based and hybrid methods show the benefit of learned priors and differentiable reconstruction layers. However, they generally depend on training data distributions, network design choices, and substantial computational resources, and their performance may degrade under domain shifts between training shapes and real degraded 3D-imaging inputs. The main RG-PSR pipeline, by contrast, is entirely deterministic and training-free, and can be applied directly to degraded point clouds without manual labels, neural network training, or ground-truth meshes. RG-PSR should, therefore, be understood as a lightweight reliability layer for Poisson-family reconstruction rather than a learned general-purpose reconstruction model.

## 3. Method

### 3.1. Overview

We propose RG-PSR, a reliability-guided enhancement framework for Poisson surface reconstruction from degraded 3D-imaging point clouds. Given an input point cloud $P={\left\{p_{i}\right\}}_{i=1}^{N}$, the goal is to reconstruct a triangular mesh *M* that is more robust to noisy normals, outliers, sparse sampling, non-uniform density, and mixed degradation.

**Figure 1:**
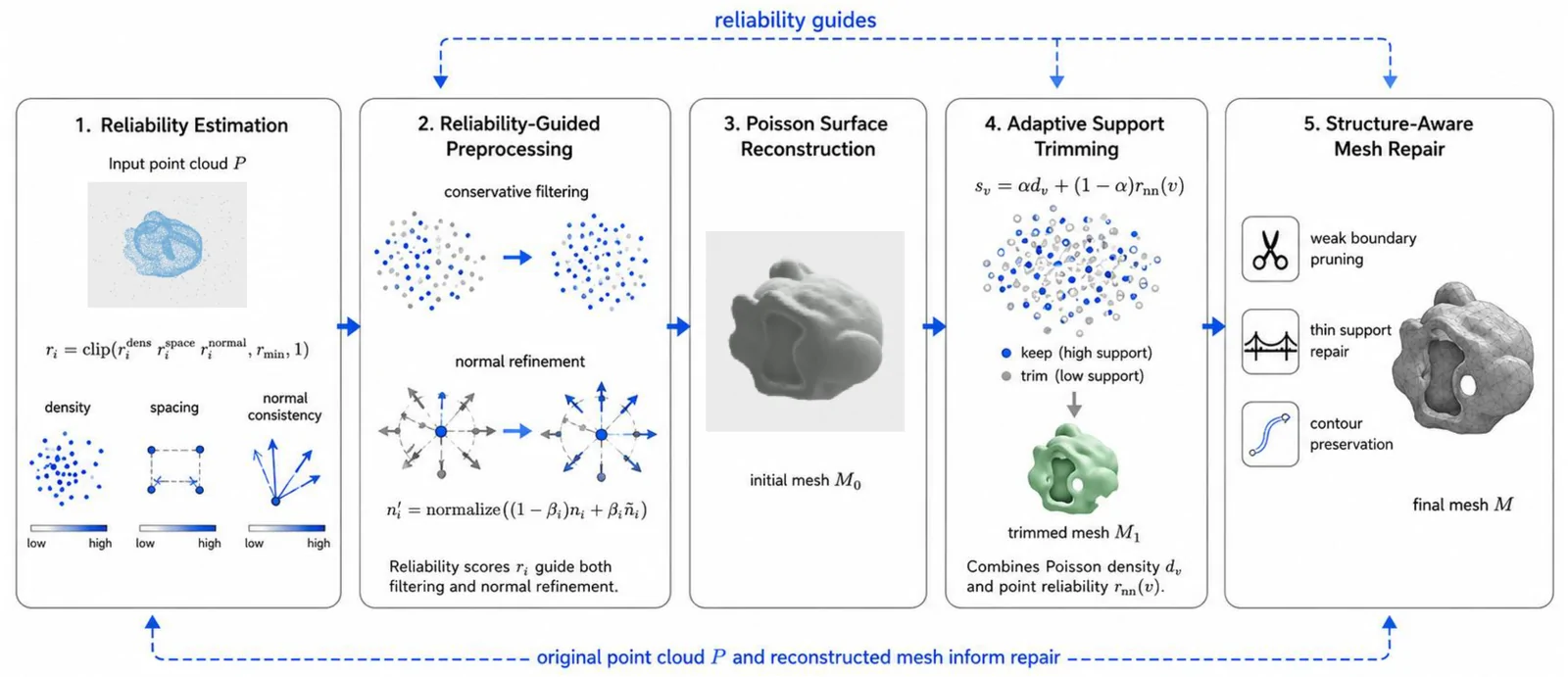
Overview of the proposed RG-PSR pipeline. Given a degraded point cloud, RG-PSR first estimates point-wise reliability from local density regularity, spacing variation, and normal consistency. The reliability scores guide preprocessing and adaptive support trimming around a Poisson reconstruction backend. A conservative structure-aware repair stage then suppresses weakly supported artifacts and produces the final triangular mesh.

As shown in Figure 1, RG-PSR consists of five main modules, and its main procedure is summarized in Algorithm 1. First, point-wise reliability is estimated from local density regularity, spacing variation, and normal consistency. Second, the reliability scores guide conservative point filtering and normal refinement. Third, Poisson surface reconstruction is applied to the refined oriented point cloud. Fourth, the reconstructed mesh is adaptively trimmed according to density-reliability support. Finally, a conservative structure-aware repair stage removes weakly supported artifacts and preserves selected input-supported structures.
**Algorithm 1** Reliability-Guided Poisson Surface Reconstruction (RG-PSR)**Require:** Degraded point cloud $P={\left\{p_{i}\right\}}_{i=1}^{N}$; parameters Θ**Ensure:** Reconstructed triangular mesh *M* 1:N←ESTIMATENORMALS(P) 2:${\left\{N_{i}\right\}}_{i=1}^{N}\leftarrow kNN\left(P,k\right)$ 3:**for** i=1 to *N* **do** 4:     $r_{i}\leftarrow R\mathrm{ELIABILITY}\left(p_{i},N_{i},N;\Theta \right)$ 5:**end for** 6:$P^{\prime }\leftarrow F\mathrm{ILTER}\left(P,\left\{r_{i}\right\}\right)$ 7:$N^{\prime }\leftarrow R\mathrm{EFINE}N\mathrm{ORMALS}\left(P^{\prime },N,\left\{r_{i}\right\},\left\{N_{i}\right\}\right)$ 8:$\left(M_{0},\rho \right)\leftarrow P\mathrm{OISSON}R\mathrm{ECON}\left(P^{\prime },N^{\prime }\right)$ 9:**for all** $v\in V(M_{0})$ **do**10:     $d_{v}\leftarrow N\mathrm{ORMALIZE}D\mathrm{ENSITY}\left(\rho_{v}\right)$11:     $j\leftarrow N\mathrm{EAREST}P\mathrm{OINT}(v,P^{\prime })$12:     $s_{v}\leftarrow \alpha d_{v}+\left(1-\alpha \right)r_{j}$13:**end for**14:$q\leftarrow A\mathrm{DAPTIVE}Q\mathrm{UANTILE}(\left\{r_{i}\right\})$15:$M_{1}\leftarrow T\mathrm{RIM}Trim\left(M_{0},\left\{s_{v}\right\},q\right)$16:$M\leftarrow S\mathrm{TRUCTURE}R\mathrm{EPAIR}(M_{1},P^{\prime },\left\{s_{v}\right\})$17:**return** *M*

For clarity, we distinguish two evaluation scopes throughout the paper. The *RG-PSR core* comprises the first four modules: reliability estimation, reliability-guided preprocessing, Poisson reconstruction, and adaptive support trimming—and produces the trimmed mesh $M_{1}$. The *complete RG-PSR pipeline* additionally applies the conditional structure-aware repair stage and produces the final mesh *M*. Unless a result is explicitly marked as “core”, RG-PSR refers to the complete pipeline.

The compact operations in Algorithm 1 correspond to the five modules described below: reliability estimation, reliability-guided preprocessing, poisson reconstruction, adaptive support trimming, and structure-aware mesh repair.

### 3.2. Reliability Estimation

The first module estimates a reliability score for each input point. The motivation is that degraded point clouds contain samples with different levels of reconstruction reliability. Isolated outliers, irregular neighborhoods, and points with unstable normals should have less influence on reconstruction, while sparse but locally consistent structures should not be removed solely because of low density.

For each point $p_{i}$, we build a *k*-nearest-neighbor neighborhood $N_{i}$, with k=32 by default. Initial normals are estimated using the same local PCA-based normal-estimation pipeline as the Poisson-family baselines. Let $h_{i}$ denote the mean neighbor distance:

(1) $$h_{i}=\frac{1}{|N_{i}|}\sum_{p_{j}\in N_{i}}{∥p_{i}-p_{j}∥}_{2}.$$

We further compute the coefficient of variation of neighbor distances:

(2) $${\mathrm{cv}}_{i}=\frac{{\mathrm{std}}_{p_{j}\in N_{i}}(∥p_{i}-p_{j}{∥}_{2})}{h_{i}+\epsilon },$$

where ϵ is a small constant for numerical stability.

The normal consistency term is computed as

(3) $$c_{i}=\frac{1}{|N_{i}|}\sum_{j\in N_{i}}\max\left(0,|n_{i}^{⊤}n_{j}|\right).$$

The absolute inner product is used because local normal signs may still contain orientation inconsistencies before refinement.

The final reliability score combines three complementary terms:

(4) $$r_{i}=\mathrm{clip}\left(r_{i}^{\mathrm{dens}}r_{i}^{\mathrm{space}}r_{i}^{\mathrm{normal}},r_{\min},1\right).$$

The density term penalizes local spacing that deviates from the global median spacing $h_{\mathrm{med}}$:

(5) $$r_{i}^{\mathrm{dens}}=\exp\left(-\frac{\left|\log\left(\left(h_{i}+\epsilon \right)/\left(h_{\mathrm{med}}+\epsilon \right)\right)\right|}{\tau_{\mathrm{dens}}}\right).$$

The spacing regularity term penalizes locally irregular neighborhoods:

(6) $$r_{i}^{\mathrm{space}}=\exp\left(-\frac{{\mathrm{cv}}_{i}^{2}}{\tau_{\mathrm{space}}^{2}}\right).$$

The normal consistency term is defined as

(7) $$r_{i}^{\mathrm{normal}}=c_{i}^{\gamma }.$$

Unless otherwise stated, we use $\tau_{\mathrm{dens}}=0.75$, $\tau_{\mathrm{space}}=0.6$, γ=2.0, and $r_{\min}=0.05$. This reliability score is deterministic, inexpensive to compute, and used as a common guidance signal for the following preprocessing and trimming modules.

Thus, the local evidence in $r_{i}$ consists of kNN distance statistics and normal dot products, not plane-fitting residuals: $r_{i}^{\mathrm{dens}}$ uses mean spacing relative to the global median, $r_{i}^{\mathrm{space}}$ uses the coefficient of variation of neighbor distances, and $r_{i}^{\mathrm{normal}}$ uses the mean absolute normal cosine. These O(k) statistics reuse the neighbor list and avoid an additional per-point eigendecomposition. The PCA-based coherence cue used for thin-structure protection does not enter Equation (4). Section 4.6 directly evaluates $r_{i}$ against geometric error, normal error, and synthetic-outlier labels.

### 3.3. Reliability-Guided Preprocessing

The second module uses the estimated reliability scores to produce a more stable oriented point cloud before Poisson reconstruction. It contains two operations: conservative reliability-guided filtering and reliability-guided normal refinement.

#### 3.3.1. Conservative Reliability-Guided Filtering

RG-PSR removes only the lowest-reliability samples before reconstruction. Let $\tau_{r}$ be a low quantile of the reliability distribution. The retained point set is

(8) $$P^{\prime }=\left\{p_{i}\in P|r_{i}\geq \tau_{r}\right\}.$$

The removal ratio is capped to avoid aggressively deleting sparse but valid structures. This step mainly suppresses isolated outliers and extremely unstable samples. Unlike density-only outlier removal, the filtering criterion combines density regularity, spacing variation, and normal consistency, making it less sensitive to local density changes alone.

#### 3.3.2. Reliability-Guided Normal Refinement

Poisson reconstruction is sensitive to normal quality. In degraded point clouds, inaccurate normals can propagate into the global implicit field and produce rough surfaces, inflated regions, or unsupported sheets. RG-PSR, therefore, refines normals by borrowing orientation information from reliable neighbors.

For each retained point $p_{i}$, we first align the sign of each neighbor normal $n_{j}$ with the center normal $n_{i}$. If $n_{i}^{⊤}n_{j}<0$, the neighbor normal is replaced by $-n_{j}$. The reliability-weighted smoothed normal is then computed as

(9) $${\tilde{n}}_{i}=\mathrm{normalize}\left(\sum_{j\in N_{i}}w_{ij}r_{j}n_{j}\right),$$

where $r_{j}$ is the reliability of neighbor $p_{j}$, and $w_{ij}$ is a Gaussian spatial weight:

(10) $$w_{ij}=\exp\left(-\frac{∥p_{i}-p_{j}{∥}_{2}^{2}}{2\sigma_{i}^{2}}\right).$$

Here, $\sigma_{i}$ is set to the median neighbor distance around $p_{i}$.

The final refined normal is obtained by blending the original and smoothed normals:

(11) $$n_{i}^{\prime }=\mathrm{normalize}\left(\left(1-\beta_{i}\right)n_{i}+\beta_{i}{\tilde{n}}_{i}\right),$$

where

(12) $$\beta_{i}=\beta_{\max}\left(1-r_{i}\right).$$

High-reliability points preserve their original normals, while low-reliability points receive stronger neighborhood correction. This design improves normal stability without globally over-smoothing well-supported regions. The output of this module is a filtered and refined oriented point cloud $\left(P^{\prime },N^{\prime }\right)$.

### 3.4. Poisson Surface Reconstruction

The third module applies Poisson surface reconstruction to the refined oriented point cloud $\left(P^{\prime },N^{\prime }\right)$. The Poisson solver estimates an implicit indicator function whose gradient is consistent with the input normal field and extracts an isosurface as an initial mesh:

(13) $$M_{0}=\left(V_{0},F_{0}\right).$$

The Poisson backend also produces per-vertex density values, which provide a measure of local support on the reconstructed surface. Although Poisson reconstruction can generate globally smooth and coherent meshes, it may still introduce weakly supported sheets, inflated boundaries, or noisy fragments under outliers, sparse sampling, and non-uniform density. Therefore, RG-PSR further prunes the initial mesh using adaptive support trimming.

### 3.5. Adaptive Support Trimming

The fourth module removes weakly supported Poisson artifacts by combining Poisson density with input-point reliability. Fixed density trimming is commonly used after Poisson reconstruction, but a single density threshold is difficult to tune across different degradation types. A valid sparse structure may have low Poisson density, while a noisy region may still have moderate density due to local point accumulation. RG-PSR, therefore, uses an adaptive density-reliability support score.

For each mesh vertex $v\in V_{0}$, let $\rho_{v}$ denote the raw Poisson density. We first normalize the density by percentile-clipped min-max normalization:

(14) $$d_{v}=\mathrm{clip}\left(\frac{\rho_{v}-Q_{0.01}\left(\rho \right)}{Q_{0.99}\left(\rho \right)-Q_{0.01}\left(\rho \right)+\epsilon },0,1\right),$$

where $Q_{0.01}\left(\rho \right)$ and $Q_{0.99}\left(\rho \right)$ are the 1st and 99th percentiles of all reconstructed vertex densities.

Let nn(v) be the nearest retained input point in $P^{\prime }$. The mesh-vertex support score is defined as

(15) $$s_{v}=\alpha d_{v}+\left(1-\alpha \right)r_{\mathrm{nn}(v)},$$

where $r_{\mathrm{nn}(v)}$ is the reliability score of the nearest input point, and α balances implicit-field support and input-point reliability.

The trimming quantile is adapted according to the mean reliability $\bar{r}$ of the retained input point cloud:

(16) $$q=\mathrm{clip}\left(q_{\min}+\lambda \left(1-\bar{r}\right),q_{\mathrm{low}},q_{\mathrm{high}}\right).$$

When the input is less reliable, trimming becomes stronger; when the input is more reliable, pruning remains conservative.

Vertices with support below the selected quantile are removed:

(17) $$V_{1}=\left\{v\in V_{0}|s_{v}\geq Q_{q}\left(S\right)\right\},$$

where $S={\left\{s_{v}\right\}}_{v\in V_{0}}$, and $Q_{q}\left(S\right)$ denotes the *q*-quantile of the support distribution. Faces incident to removed vertices are discarded. Small isolated components with insufficient support are also removed. The resulting mesh is denoted as

(18) $$M_{1}=\left(V_{1},F_{1}\right).$$

### 3.6. Structure-Aware Mesh Repair

The fifth module performs conservative structure-aware mesh repair. This stage uses only the degraded input point cloud and the reconstructed mesh. It does not query the ground-truth mesh during inference. Its purpose is not semantic completion, but suppression of weakly supported artifacts and preservation of selected input-supported structures.

#### 3.6.1. Weak Boundary Pruning

After adaptive trimming, RG-PSR compares reconstructed mesh regions with reliable input support. For each mesh vertex *v*, we compute the nearest-point distance to the retained input point set:

(19) $$\delta_{v}=\min_{p_{i}\in P^{\prime }}{∥v-p_{i}∥}_{2}.$$

Vertices or boundary regions with both large $\delta_{v}$ and low support score $s_{v}$ are treated as likely artifacts. These regions are pruned conservatively to suppress floating sheets, skirt-like boundaries, and weak surface fragments. This operation is subtractive: it starts from the Poisson-family reconstruction and removes poorly supported geometry rather than rebuilding the mesh from scratch.

#### 3.6.2. Thin Support Repair

For thin or support-like structures, Poisson reconstruction may produce disconnected, inflated, or overly smoothed geometry. RG-PSR applies local repair only when sufficient input evidence exists. Candidate regions are detected from the input point cloud using density-based local clustering (DBSCAN) [37] and geometric extent statistics. A candidate cluster must satisfy three conditions: it should be close to a weak or missing mesh region, form a coherent spatial cluster, and show an elongated local distribution.

For each candidate cluster *C*, we compute a local principal component analysis. Let $\lambda_{1}\geq \lambda_{2}\geq \lambda_{3}$ be the eigenvalues of the cluster covariance matrix. The elongation score is defined as

(20) $$e\left(C\right)=\frac{\lambda_{1}}{\lambda_{2}+\lambda_{3}+\epsilon }.$$

Clusters with sufficient point support and high elongation are considered thin-structure candidates. When enough evidence exists, RG-PSR fits a simple local support mesh within the spatial extent of the observed input points and connects it to nearby reliable body regions. Because this operation uses a geometric prior, it is reported as structure-aware repair rather than a universal thin-structure reconstruction solution.

#### 3.6.3. Contour Preservation and Local Smoothing

For boundary-like regions with strong contour evidence, RG-PSR applies constrained contour preservation. Boundary-band vertices are displaced toward a locally fitted contour with clipped displacement:

(21) $$v^{\prime }=v+\eta \;\mathrm{clip}\left(\Pi \left(v\right)-v,\Delta_{\max}\right),$$

where Π(v) denotes the projected position on the local contour, η is a blending factor, and $\Delta_{\max}$ limits the maximum displacement.

Finally, light local smoothing is applied to noisy or repaired regions. The smoothing is restricted to low-confidence or repaired regions and avoids high-confidence body regions. This reduces high-frequency artifacts while preserving the overall reconstructed shape. The final output is the triangular mesh *M*.

Table 1 summarizes the fixed parameters used by the structure-aware repair stage. All repair parameters are selected once on the validation split and are not tuned for individual test shapes during evaluation. Support repair is triggered only when the fixed candidate tests are satisfied; no repaired region is manually selected from an output mesh.

### 3.7. Complexity and Implementation

The RG-PSR core adds reliability estimation and conservative filtering, reliability-guided normal refinement, nearest-neighbor mesh-support queries, and adaptive trimming on top of the standard Poisson backend. The complete pipeline additionally applies conditional structure-aware repair. All modules are deterministic and require no neural network training; nearest-neighbor operations are accelerated with spatial indexing structures such as KD-trees.

For the core pipeline, the main additional computational cost comes from neighborhood search, normal refinement, and mesh-support queries. The method remains suitable for normalized object-level reconstruction on standard desktop hardware. All parameters are selected on a validation split and then fixed for the test set; no per-sample tuning is used.

Hardware, software, and core-pipeline cost.

All methods run on CPU only: Intel Core i7-8700 (6 cores/12 threads, 3.20 GHz), 64 GB RAM, Ubuntu 5.15, Python 3.9, and Open3D 0.19. Wall-clock times are averaged over the fully crossed 75-case pool at Poisson depth 8. As Table 2 shows, the shared Poisson solve dominates both methods. The RG-PSR core takes 6.43 s, compared with 6.08 s for Poisson + Trim, corresponding to a core-only overhead of 0.35 s (5.8%). The conditional structure-aware repair stage was not instrumented in these timing runs. Therefore, every runtime and overhead value reported in this paper refers exclusively to the RG-PSR core, and no complete-pipeline runtime or efficiency claim is made.

All core-pipeline runtimes used in the module-cost and ablation analyses were remeasured under this common environment. Earlier Poisson-family timings that could not be reproduced were replaced; the sensitivity-study timings are used only for within-sweep core-cost comparison.

Memory and scaling with point count.

Table 3 reports isolated-process measurements on bunny resampled from 2000 to 50,000 points at depth 8. The added reliability and support queries use spatial indexing and require O(nlogn) time and O(n) storage.

Across this 25× increase in point count, peak RSS rises from 641/642 MB to 833/834 MB for Poisson/RG-PSR core, while runtime rises from 6.4/7.0 s to 14.5/19.1 s. Thus, the tested object-level regime shows moderate memory growth and no change in asymptotic complexity for the core pipeline.

## 4. Experiments

### 4.1. Experimental Setup

#### 4.1.1. Datasets

We evaluate RG-PSR on a controlled benchmark composed of three dataset groups that cover scanned object meshes, category-level object meshes, and CAD-style stress tests. The first group, denoted *Open3D/Stanford*, contains 5 Stanford/Open3D-style meshes, bunny and armadillo, together with three additional Open3D model assets (knot, monkey, and damaged helmet). The second group is a ModelNet10 subset containing 4 object meshes (two chairs and two bathtubs) from multiple categories. The third group is a deterministic procedural CAD stress-test set containing 6 hard-edge and thin-structure shapes. In total, the strict surface-baseline benchmark contains these same 15 base shapes.

The main comparison contains 52 cases: nine shapes are tested under all five degradations (45 cases), while bunny, armadillo, and the four ModelNet10 shapes contribute seven representative test cases in total. Across seven methods, this gives 364 reconstructions. The ablation uses the fully crossed 75-case design; the reliability-validity study uses all 15 shapes under the variants specified in Section 4.6. All meshes are cleaned by removing duplicated and degenerate elements, centered at the origin, and normalized to unit bounding-box diagonal. The purpose of the benchmark is controlled degradation analysis rather than large-scale category generalization.

For each ground-truth mesh, we sample input point clouds and apply deterministic degradations with fixed random seed 42. The evaluated degradation variants include medium Gaussian noise, sparse sampling, non-uniform density, outlier contamination, and mixed degradation. These variants represent the main failure modes targeted by RG-PSR: inaccurate normals, low local support, irregular density, and weakly supported outlier-induced surfaces.

#### 4.1.2. Compared Methods

We compare RG-PSR with representative classical surface-reconstruction baselines: Poisson surface reconstruction, Screened Poisson reconstruction, Poisson with fixed density trimming, Alpha Shape, Ball Pivoting Algorithm (BPA), and Greedy Projection Triangulation (GP3). All methods use the same input point cloud for each case. Methods that require normals use the same initial normal-estimation pipeline. Parameters are selected on a validation split and then fixed globally; no method is tuned per test sample. The primary claim is evaluated within the Poisson family: Poisson, Screened Poisson, Poisson + Trim, and RG-PSR. Alpha Shape, BPA, and GP3 are included to position RG-PSR relative to broader classical reconstruction methods.

#### 4.1.3. Parameter Selection and Data-Splitting Protocol

Two parameter-selection procedures are used. First, classical-baseline parameters and the surface-refinement settings are selected on eight ModelNet10 case-level validation examples. The exact cases are bathtub_bathtub_0107 with clean, noise_medium, and nonuniform_strong; bathtub_bathtub_0108 with clean, noise_medium, nonuniform_strong, and outliers_5; and bathtub_bathtub_0109 with clean. This set is generated deterministically from the manifest: eligible variants are filtered, exact dataset–shape–variant triples used in the main benchmark are removed, the remaining cases are sorted by dataset, shape identifier, and variant, and the first eight are selected. The split is, therefore, disjointed at the exact-case level but not at the shape level; bathtub_bathtub_0107 and bathtub_bathtub_0109 also occur in the final benchmark under other degradations. The selection is not random or stratified, and we state this limitation explicitly.

Second, the RG-PSR core parameters (trimming strength, $\beta_{\max}$, and *k*) are swept on bunny and chair_0890 under all five degradations; procedural CAD is entirely excluded from this sweep. The selected values are then fixed globally. Because bunny and chair_0890 also appear in the final tables, this protocol has shape-level overlap, including one exact overlap (bunny/*outliers_5*). Fully shape-disjoint validation is, therefore, left to future benchmark work. The seven representative cases described in Section 4.1.1 refer to the final test composition and are unrelated to the eight case-level validation examples listed above.

#### 4.1.4. Metrics

We use metrics that correspond directly to the reported comparisons. Geometric accuracy is computed from triangular surfaces rather than raw mesh vertices. For each reconstructed mesh *M* and ground-truth mesh *G*, we uniformly sample two area-weighted point sets $S_{M}$ and $S_{G}$ from triangular faces, with $10^{5}$ samples per mesh. Let $D_{G}$ be the ground-truth bounding-box diagonal. We use the standard symmetric Chamfer-$L_{1}$ distance:

(22) $${\mathrm{CD}}_{L_{1}}\left(M,G\right)=\frac{1}{2D_{G}}\left(\frac{1}{|S_{M}|}\sum_{x\in S_{M}}\min_{y\in S_{G}}{∥x-y∥}_{2}+\frac{1}{|S_{G}|}\sum_{y\in S_{G}}\min_{x\in S_{M}}{∥y-x∥}_{2}\right).$$

F-score is computed from precision and recall:

(23) $$F_{\tau }=\frac{2P_{\tau }R_{\tau }}{P_{\tau }+R_{\tau }},$$

where

(24) $$P_{\tau }=\frac{1}{|S_{M}|}\sum_{x\in S_{M}}1\left[\min_{y\in S_{G}}{∥x-y∥}_{2}\leq \tau D_{G}\right],\;R_{\tau }=\frac{1}{|S_{G}|}\sum_{y\in S_{G}}1\left[\min_{x\in S_{M}}{∥y-x∥}_{2}\leq \tau D_{G}\right].$$

We report $F_{0.01}$ for the main baseline comparisons and use $F_{0.02}$ in the parameter-sensitivity and ablation analyses. Because $F_{\tau }$ can mask precision–recall asymmetry, Table 4 also reports $P_{0.01}$ and $R_{0.01}$, where recall measures ground-truth coverage/completeness.

To evaluate mesh coherence, we report the number of connected components computed on the triangle adjacency graph. We also report the largest-component ratio:

(25) $$\mathrm{LCR}=\frac{\max_{c}\left|F_{c}\right|}{\left|F\right|},$$

where $F_{c}$ is the set of faces in connected component *c*, and *F* is the full face set. A lower component count and a higher largest-component ratio indicate a more coherent object-level mesh.

To measure weakly supported reconstructed geometry, we report ${\mathrm{Artifact}}_{0.02}$:

(26) $${\mathrm{Artifact}}_{0.02}=1-P_{0.02}.$$

This metric measures the fraction of reconstructed surface samples that are farther than $0.02D_{G}$ from the ground-truth surface. It is used as a support-error indicator rather than a complete measure of mesh quality.

All reported runtime values refer to the RG-PSR core and exclude conditional structure-aware repair. The complete pipeline is evaluated for reconstruction quality, but no complete-pipeline runtime or efficiency claim is made. For the LiDAR-like scanning case study, no ground-truth mesh is available. Therefore, that subsection uses a separate held-out point-to-mesh evaluation protocol, including mean held-out distance and coverage at fixed metric thresholds.

### 4.2. Effectiveness of RG-PSR for Poisson-Family Reconstruction

The preceding baseline comparison shows that local triangulation methods such as BPA and GP3 can achieve very strong point-wise accuracy under favorable sampling conditions. However, the primary goal of RG-PSR is not to replace all classical reconstruction methods, but to improve the robustness of Poisson-family reconstruction under degraded inputs. We, therefore, isolate the Poisson-family comparison in this subsection to directly evaluate the intended claim: whether reliability-guided filtering, normal refinement, adaptive trimming, and conservative postprocessing improve Poisson reconstruction without changing its global implicit reconstruction backbone.

Table 5 reports the Poisson-family comparison on the three dataset groups. Relative to Poisson + Trim, RG-PSR improves both Chamfer-$L_{1}$ and $F_{0.01}$ on Open3D/Stanford, ModelNet10, and Procedural CAD. It also reduces ${\mathrm{Artifact}}_{0.02}$ from 0.3090 to 0.2632, indicating fewer weakly supported reconstructed surface regions. These results support the main claim that RG-PSR improves Poisson-family reconstruction under degraded point-cloud inputs. The reconstruction-quality results in this subsection correspond to the complete RG-PSR pipeline, including conditional structure-aware repair. Core-only computational cost is analyzed separately in Section 3.7; because the repair stage was not timed, no complete-pipeline runtime or efficiency claim is made.

Relative to Poisson + Trim, RG-PSR reduces Chamfer-$L_{1}$, ${\mathrm{Artifact}}_{0.02}$, and component count by 21.1%, 14.8%, and 35.7%, respectively, while improving $F_{0.01}$ by 3.3%. Relative to vanilla Poisson, the corresponding changes are 49.1%, 32.0%, 35.6%, and 11.6%. The gains are also case-consistent: RG-PSR improves Chamfer-$L_{1}$ in 42/52 cases and $F_{0.01}$ in 41/52. Chamfer-$L_{1}$ standard deviation is 0.0161, versus 0.0201 for Poisson + Trim and 0.0297 for Poisson.

**Figure 2:**
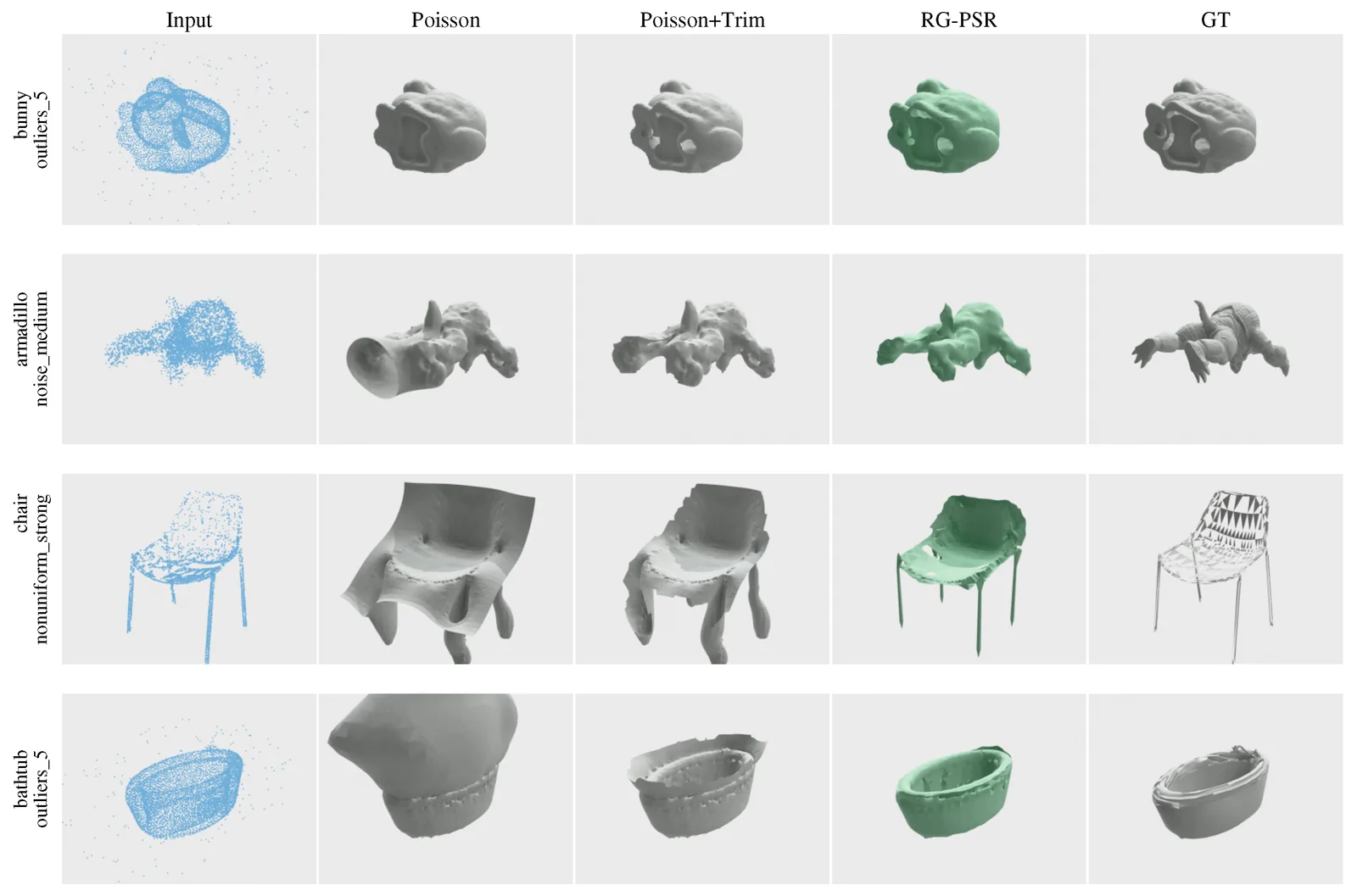
RG-PSR qualitative comparison. Reconstructed results are rendered as shaded triangular meshes, not sampled point clouds.

Figure 2 shows representative shaded triangular-mesh renderings. The examples highlight common Poisson-family failure modes such as inflated boundaries, unsupported sheets, rough surfaces, and damaged thin structures. RG-PSR reduces many of these artifacts while preserving the global mesh character of Poisson reconstruction.

### 4.3. Comparison with Classical Surface-Reconstruction Baselines

After isolating the Poisson-family comparison, we further compare RG-PSR with broader classical surface-reconstruction methods. This subsection is not intended to rank all methods by a single metric; instead, it contrasts point-wise geometric accuracy with global mesh coherence.

Table 6 gives a compact comparison with broader classical surface-reconstruction methods. Since the previous subsection already isolates the Poisson-family comparison, this table reports only two overall point-wise metrics and two mesh-coherence metrics. The local triangulation methods, especially BPA and GP3, achieve the best point-wise Chamfer and F-score values because they closely follow the input samples. However, they produce many disconnected components and have low largest-component ratios. In contrast, RG-PSR achieves the lowest component count and the highest largest-component ratio among all seven methods, showing its main all-method advantage: coherent global meshing behavior.

The compact comparison in Table 6 shows two different regimes. GP3 gives the best point-wise accuracy, with the lowest overall Chamfer-$L_{1}$ (0.0056) and the highest overall $F_{0.01}$ (0.8390), but it also produces the most fragmented output, with 176.19 connected components and a largest-component ratio of only 0.6911. BPA shows a similar pattern: strong point-wise accuracy but 85.81 components and a largest-component ratio of 0.6245. By contrast, RG-PSR has higher point-wise error than these local triangulation methods, but it produces the most coherent mesh structure, with the fewest components (16.25) and the highest largest-component ratio (0.9872). This supports the intended interpretation that RG-PSR trades some local interpolation accuracy for substantially improved global mesh coherence.

**Figure 3:**
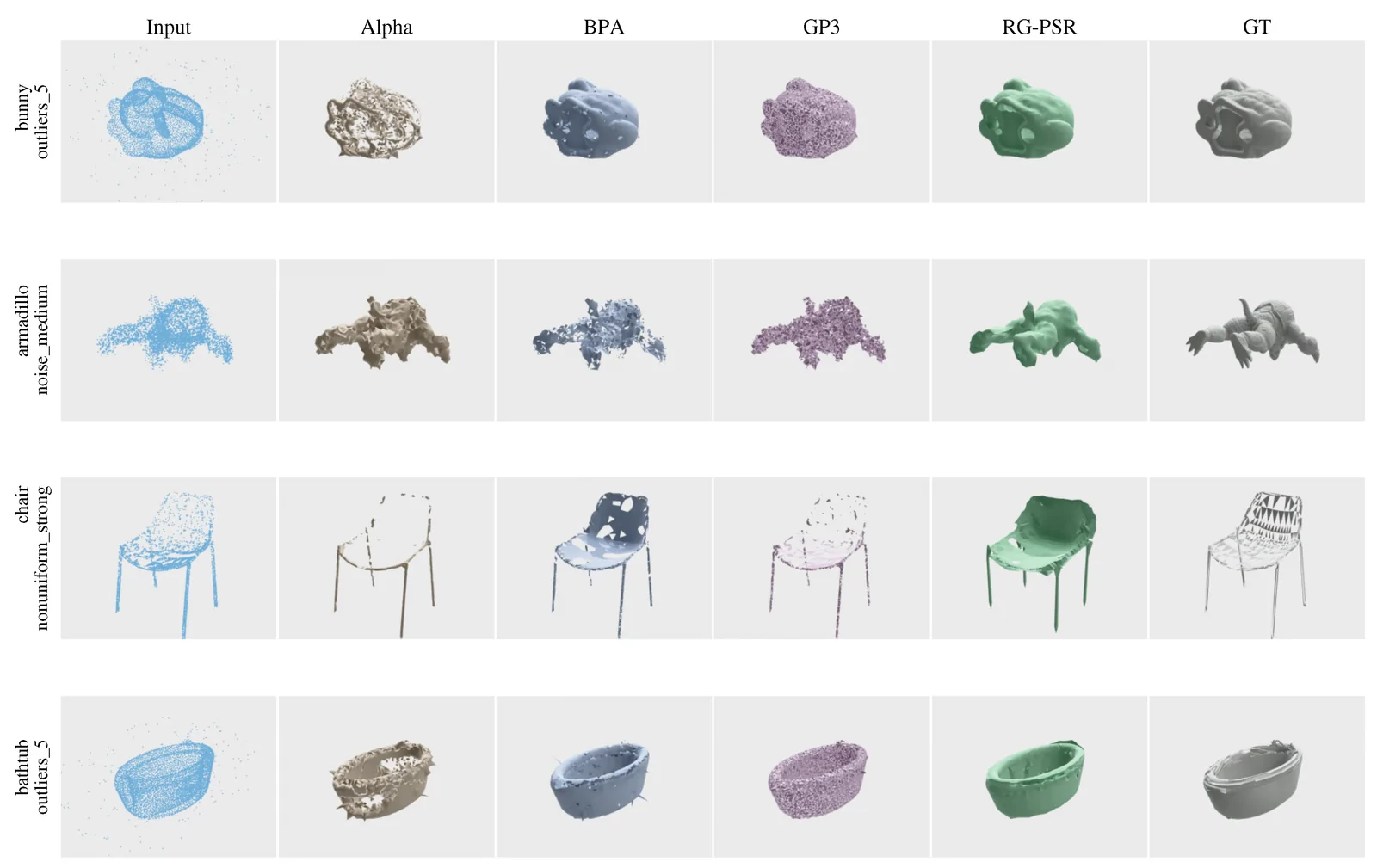
Classical surface-reconstruction baseline comparison excluding Poisson-family intermediate variants. BPA and GP3 can achieve strong point-wise accuracy but often generate open or fragmented meshes, while RG-PSR preserves a more coherent global reconstruction.

Figure 3 provides a qualitative view of the same trade-off. Alpha Shape, BPA, and GP3 often follow the observed samples closely, but their outputs contain holes, open boundaries, or many small disconnected pieces, especially under outliers, noise, and non-uniform sampling. The RG-PSR reconstructions are smoother and less locally interpolatory, but they remain visually more coherent as single reconstructed surfaces. This visual evidence is consistent with Table 6: local triangulation methods are strong point-wise baselines, whereas RG-PSR is stronger when global mesh coherence is the target.

### 4.4. Parameter Selection and Sensitivity

**Figure 4:**
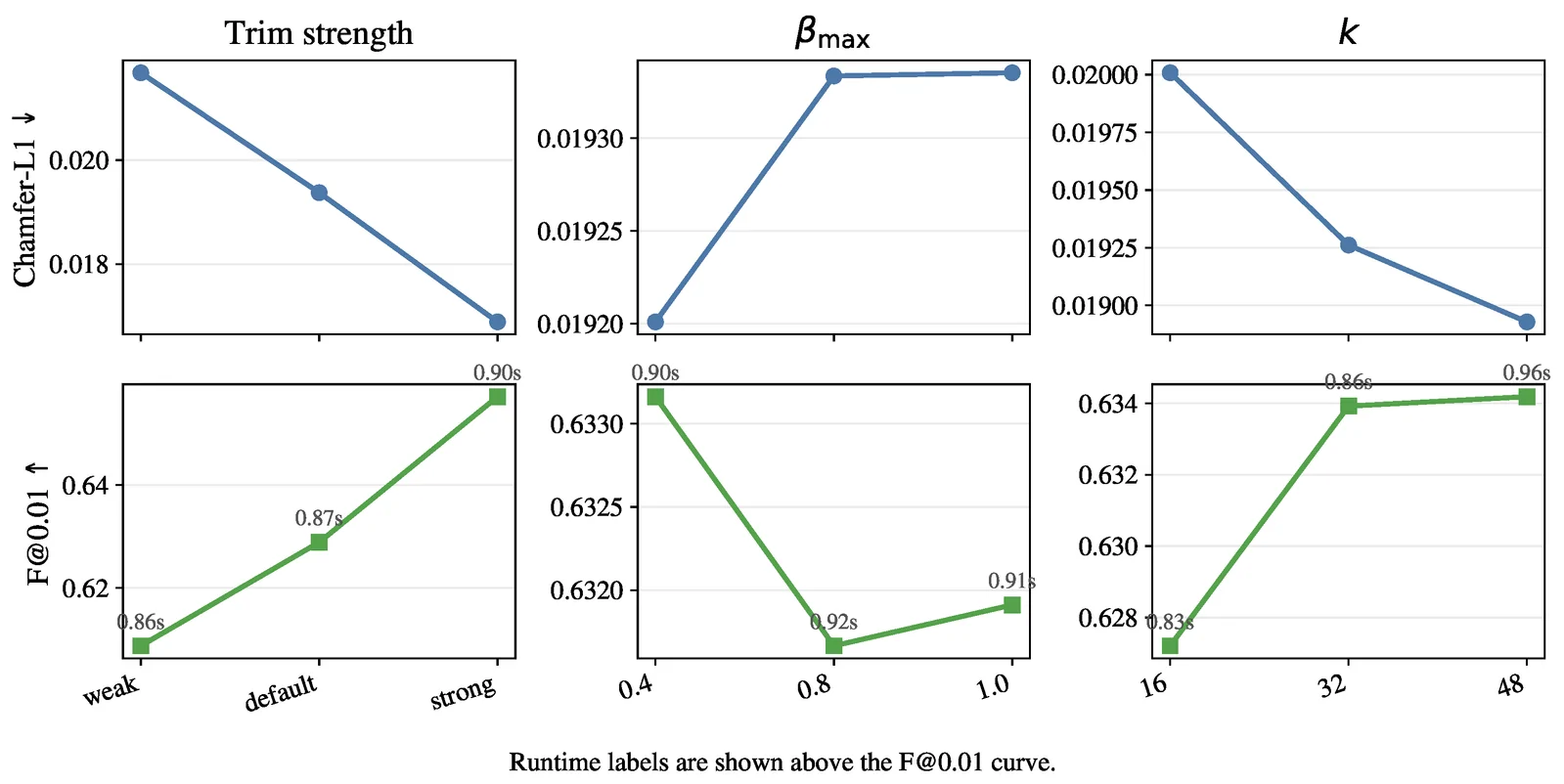
RG-PSR parameter sensitivity on the validation split. The three columns vary trimming strength, normal-refinement strength $\beta_{\max}$, and neighborhood size *k*, respectively. The top row plots Chamfer-$L_{1}$ and the bottom row plots $F_{0.01}$; core-runtime labels, excluding conditional structure-aware repair, are shown above the $F_{0.01}$ curves. ↑ indicates that higher values are better, and ↓ indicates that lower values are better.

We evaluate the sensitivity of RG-PSR to three important parameters: trimming strength, normal-refinement strength $\beta_{\max}$, and reliability-neighborhood size *k*. The validation split covers Open3D/Stanford and ModelNet10 cases (see Section 4.1.3 for the exact composition and overlap). Table 7 reports the numerical results, while Figure 4 visualizes the same trends for the three parameter sweeps. In each sweep, one parameter is varied while the others are fixed, and the final method uses a single global parameter setting rather than per-test-shape tuning. The timing values in this sensitivity study measure the core pipeline only and are used exclusively for within-sweep comparison; the freshly remeasured absolute core runtime is reported in Table 2.

The visualization shows that trimming strength has the clearest effect: moving from weak to strong trimming decreases Chamfer-$L_{1}$ from 0.0217 to 0.0169 and increases $F_{0.01}$ from 0.6087 to 0.6572, with only a small core-runtime increase. This confirms that suppressing weakly supported Poisson regions is the dominant source of improvement on the validation split, although overly aggressive trimming can still damage thin structures in difficult cases. The $\beta_{\max}$ sweep is nearly flat, indicating that the method is not highly sensitive to the exact normal-refinement blend weight. Increasing *k* from 16 to 48 slightly improves Chamfer-$L_{1}$ and $F_{0.01}$, but also increases core runtime from 0.83 s to 0.96 s; therefore, k=32 provides a practical accuracy–cost trade-off.

**Figure 5:**
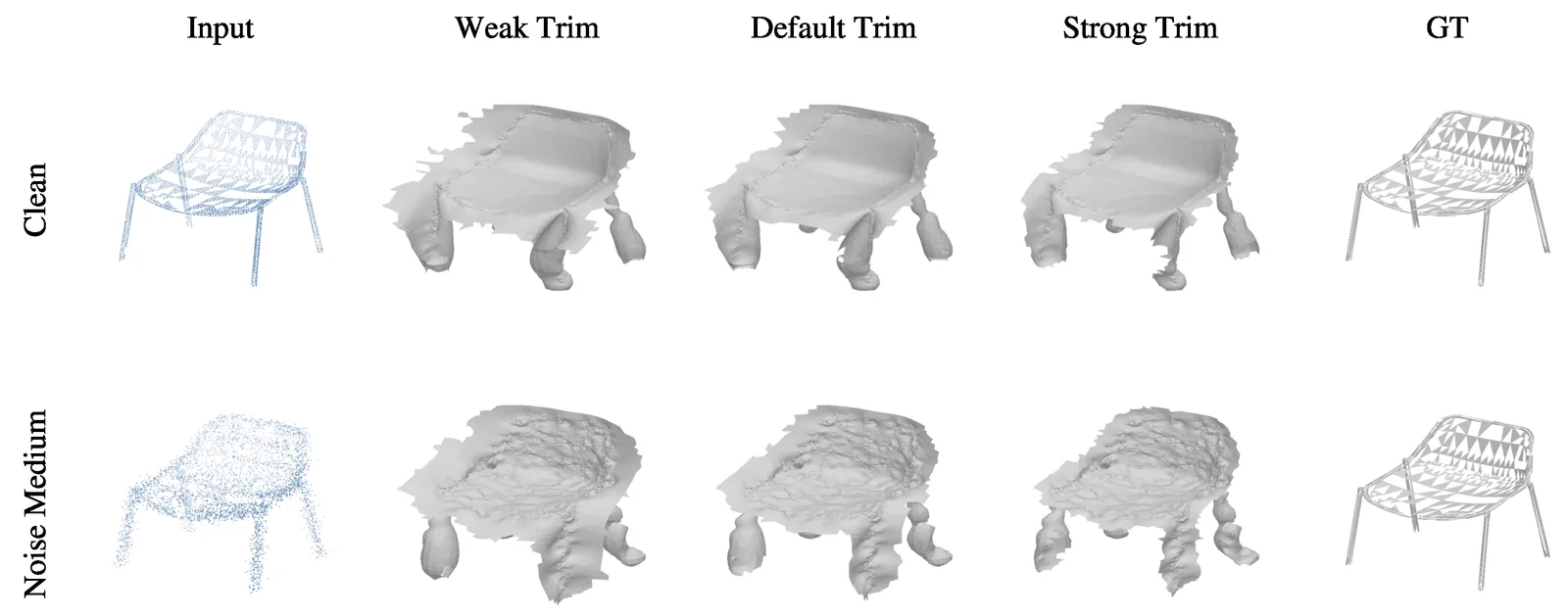
Qualitative reconstruction results under different trimming strengths on a representative non-uniform ModelNet10 chair case. Weak, default, and strong trimming use the same RG-PSR pipeline except for the adaptive trimming parameters.

Because trimming strength produces the largest quantitative change, Figure 5 further visualizes the corresponding reconstructions on the ModelNet10 *chair_chair_0890* shape under clean and medium-noise inputs. Weak trimming is conservative and leaves more low-support Poisson sheets, especially in the noisy case. Default trimming removes many unsupported regions while retaining more of the chair body and leg support, giving a visually balanced reconstruction. Strong trimming removes additional weak regions, but can also reduce sparse or noisy support. This illustrates why the trimming parameter is selected globally on the validation split rather than tuned per test shape.

### 4.5. Ablation Study

We re-ran the ablation on all 15 shapes under all five degradations (75 cases) at Poisson depth 8. This resolves the earlier Table 5 discrepancy caused by a smaller case pool and depth 7. The resulting Poisson Chamfer-$L_{1}$ (0.0333) is close to the 52-case main-table value (0.0338). This quantitative ablation evaluates the RG-PSR core only; the conditional structure-aware repair stage is excluded from every row. Accordingly, the last row represents the complete set of core modules rather than the complete RG-PSR pipeline. Its Chamfer-$L_{1}$ of 0.0179, therefore, differs from the headline value of 0.0172 obtained by the complete pipeline.

Table 8 shows that adaptive trimming provides the largest single-module gain and that the RG-PSR core gives the best overall core-only results. Chamfer-$L_{1}$ standard deviation also decreases from 0.0279 for Poisson to 0.0146 for the RG-PSR core.

**Figure 6:**
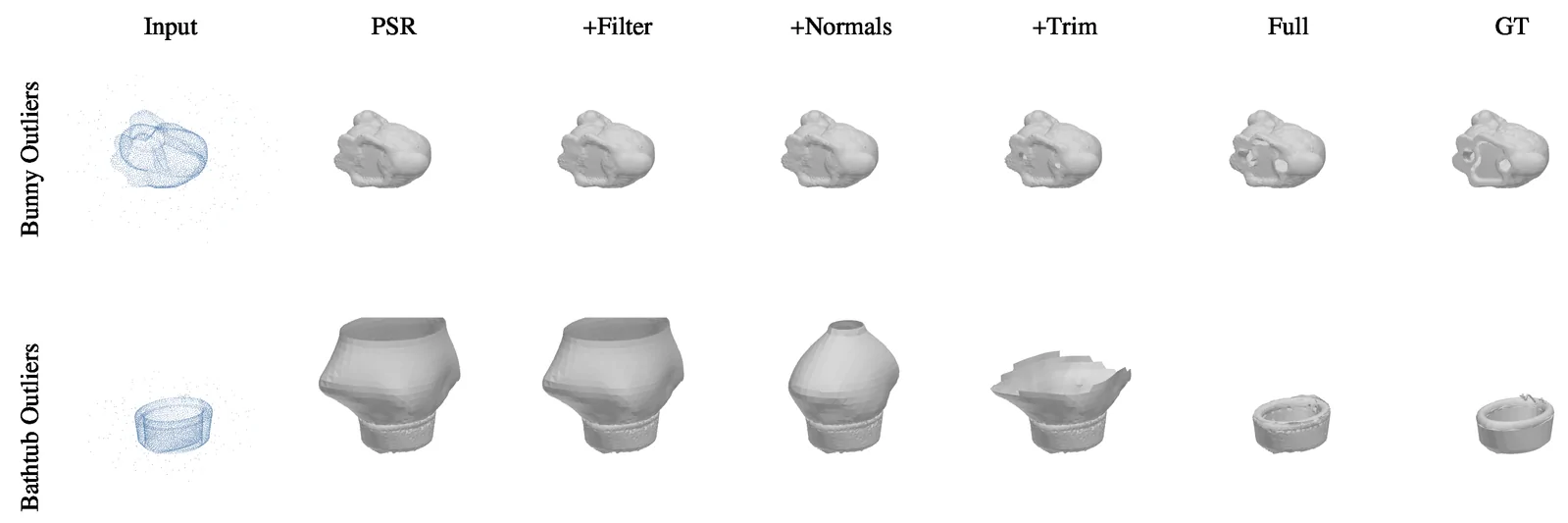
Qualitative module comparison on representative outlier-contaminated bunny and bathtub cases. The final column shows the complete RG-PSR pipeline, including conditional structure-aware repair; Table 8 reports the core-only quantitative ablation.

Figure 6 provides a qualitative module comparison on two representative outlier-contaminated cases from Figure 3: the Stanford bunny and a ModelNet10 bathtub. The preprocessing-only variants remain close to vanilla Poisson and mainly stabilize the input support, whereas adaptive trimming visibly removes weakly supported Poisson sheets and boundary artifacts. The final column shows the complete RG-PSR pipeline, including conditional structure-aware repair, and serves as a qualitative reference to the main pipeline. Table 8, by contrast, is a core-only quantitative ablation.

### 4.6. Reliability-Score Validity

We directly validate $r_{i}$ on all 15 shapes. For *noise_medium*, *nonuniform_strong*, and *mixed_medium* (45 cases; 166,050 points), point error is the normalized distance to the ground-truth surface and normal error is the angle to the nearest ground-truth surface normal. For *outliers_5*, the recorded injected-point indices provide exact outlier labels. To avoid confounding by different error scales across cases, Spearman’s ρ is computed within each case and combined with a Fisher-*z*-weighted average.

Table 9 shows that reliability is consistently associated with lower normal error ($\rho (r_{i},-e_{n})=0.462$, positive in all 45 cases), but only weakly with lower point error ($\rho (r_{i},-e_{p})=0.025$). Using $1-r_{i}$ for outlier detection yields AUROC 0.940±0.040 and AUPRC 0.832±0.088 across 15 shapes; mean reliability is 0.133 for outliers and 0.547 for inliers.

Thus, $r_{i}$ is supported as a neighborhood-normal reliability and outlier cue, not as a general estimator of per-point coordinate noise.

### 4.7. LiDAR/Scanning Case Study

We include a lightweight LiDAR-like scanning case study to illustrate a practical application scenario under sparse, anisotropic, and incomplete point distributions. Specifically, we construct three deterministic LiDAR-like accumulated blocks generated by the provided scanning script. The blocks are stored in metric scale, cropped to local regions, and split into 80% reconstruction points and 20% held-out evaluation points. These point clouds contain occlusion-like missing regions, uneven height distributions, and sparse local structures, making them qualitatively different from normalized object meshes. Since no ground-truth mesh is available, we report held-out point-to-mesh distance and coverage in Table 10. This experiment is an application case study rather than a fully supervised LiDAR surface benchmark.

**Figure 7:**
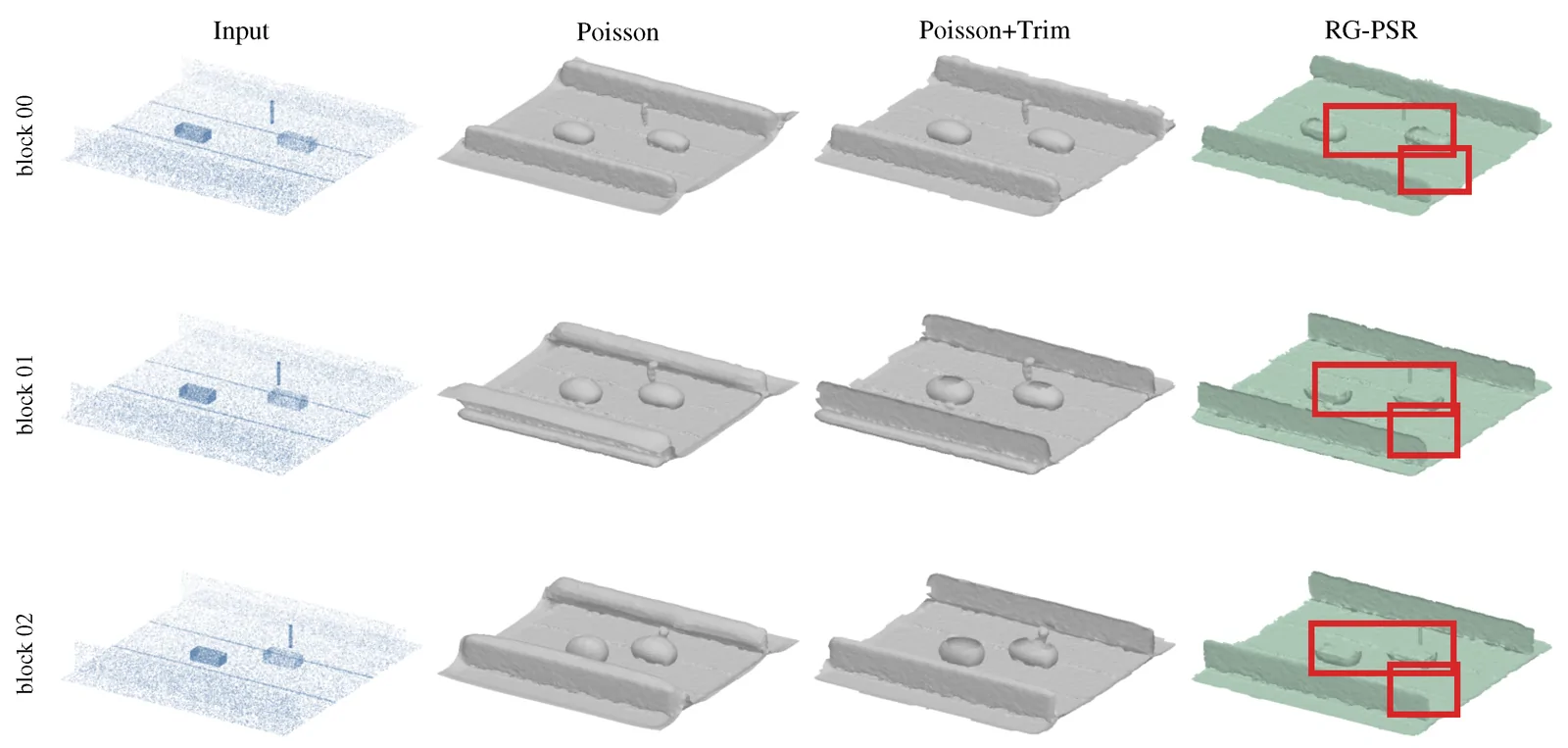
LiDAR/scanning case study meshes. Red boxes highlight local regions with weakly supported artifact geometry. RG-PSR preserves comparable held-out coverage while reducing such artifacts. Evaluation uses held-out point-to-mesh metrics because no ground-truth mesh is available.

Figure 7 visualizes the three LiDAR-like accumulated blocks used in the case study. The input point clouds are sparse and anisotropic, with large ground-like regions, vertical structures, and small object-like supports. Vanilla Poisson reconstruction tends to generate broad smooth surfaces and weakly supported sheets around sparse regions. Fixed density trimming removes part of these unsupported regions, but may still retain rough boundary sheets or unstable local structures. In contrast, RG-PSR produces comparable global coverage while reducing weakly supported geometry. The red boxes highlight local regions where the reliability-guided support check affects small scan-supported structures and nearby weak surfaces. This visualization should be interpreted as qualitative evidence for artifact suppression and support preservation under sparse LiDAR-like inputs, rather than as a fully supervised LiDAR surface benchmark.

### 4.8. Discussion

The experiments support the intended positioning of RG-PSR as a reliability-guided enhancement of Poisson-family surface reconstruction. In the isolated Poisson-family comparison, RG-PSR improves Chamfer-$L_{1}$, $F_{0.01}$, and ${\mathrm{Artifact}}_{0.02}$ over vanilla Poisson, Screened Poisson, and fixed density trimming. These results indicate that point reliability, normal refinement, adaptive density-reliability trimming, and conservative postprocessing are useful for reducing weakly supported Poisson artifacts under degraded point-cloud inputs.

The comparison with broader classical baselines clarifies the trade-off between point-wise accuracy and global mesh coherence. Local triangulation methods such as BPA and GP3 can achieve lower Chamfer distance and higher F-score because they closely follow the observed samples. However, they also produce more fragmented outputs, with many connected components and lower largest-component ratios. In contrast, RG-PSR does not aim to be the best point-wise interpolating method; its main advantage is producing a more coherent Poisson-family mesh with fewer disconnected components and a higher largest-component ratio. Therefore, the appropriate conclusion is not universal superiority over all reconstruction methods, but improved robustness and coherence within a Poisson reconstruction pipeline.

Several limitations remain. First, the reliability score uses a global median spacing term, which may be biased when a point cloud contains strongly different density modes; Section 4.6 also shows that it captures normal consistency and outliers better than raw coordinate error. Second, the RG-PSR core introduces additional runtime due to neighborhood search, normal refinement, and support queries. The remeasured core-only overhead is 5.8% relative to Poisson + Trim. Because the conditional structure-aware repair stage was not timed, the runtime of the complete pipeline remains to be quantified (Section 3.7). Third, the structure-aware repair stage is conservative and may over-smooth details or over-trim very thin structures when input support is ambiguous. Finally, the LiDAR-like case study is only a preliminary held-out evaluation without ground-truth meshes, and the procedural CAD set is a controlled stress test rather than a large external benchmark. Future work will extend the reliability framework to larger CAD datasets, sparse outdoor scans, and more robust thin-structure handling, including integration with iterative orientation methods such as iPSR [31].

Pitfalls of prioritizing high-confidence points.

Filtering is quantile-capped, and trimming becomes conservative as mean reliability increases, reducing but not eliminating the risk of holes in sparse valid regions. Table 4 shows that RG-PSR retains high recall (0.797), whereas strong trimming can erode thin structures despite improving aggregate Chamfer-$L_{1}$ and F-score (Figure 5). We, therefore, use the default rather than strong trimming setting.

Sharp features versus global smoothing.

Because $\beta_{i}\;\to \;0$ as $r_{i}\;\to \;1$, well-supported sharp features receive little normal smoothing; the procedural-CAD set tests this behavior. Nevertheless, the underlying Poisson kernel still rounds sharp creases, and RG-PSR cannot recover a crease that is itself sparsely or noisily sampled. A feature-preserving Poisson backend is, therefore, a natural extension.

## 5. Conclusions

This paper presented the complete RG-PSR pipeline, a reliability-guided framework for improving Poisson-family surface reconstruction from degraded point clouds. The method estimates point-wise reliability from local density regularity, spacing variation, and normal consistency, and propagates this reliability through conservative filtering, normal refinement, adaptive density-reliability trimming, and structure-aware mesh repair. The main pipeline is deterministic and training-free, requiring no manual labels, neural network training, or ground-truth geometry during reconstruction. Experiments on three dataset groups show that RG-PSR improves Poisson-family reconstruction under degraded inputs. Compared with vanilla Poisson, Screened Poisson, and fixed density trimming, it reduces geometric error, suppresses weakly supported artifacts, and improves global mesh coherence. At the same time, local triangulation methods such as BPA and GP3 remain strong in point-wise geometric accuracy under favorable sampling conditions, and can outperform RG-PSR in Chamfer distance and F-score. Thus, RG-PSR should be understood as a practical reliability layer for Poisson reconstruction rather than a universal replacement for all surface-reconstruction methods. Future work will extend the reliability framework to broader reconstruction backends, larger external CAD datasets, sparse outdoor scans, and more robust handling of complex thin structures.

## Figures and Tables

**Table 1:** Fixed parameters for the structure-aware repair stage. Distances are expressed in normalized object coordinates unless noted otherwise.

Component	Parameter	Value
Boundary support pruning	reliable support quantile ($q_{\mathrm{rel}}$)	0.45
	support distance scale ($\kappa_{\delta }$)	2.3 local spacings
	support threshold range	(0.16)–(0.38)
	maximum removed fraction	0.30
Thin-support detection	lower-height fraction	0.58
	DBSCAN (ϵ,minPts)	(0.022, 10)
	minimum cluster size ($n_{\min}$)	60 points
	minimum length ($l_{\min}$)	0.18
	elongation threshold ($e_{\min}$)	3.0
Thin-support fitting	radius percentile and clip	(45%×0.85, [0.0045, 0.0095])
	cylinder angular resolution	28
Thin-support connection	edge search radius	($\max(0.080,3.5r_{\mathrm{socket}})$)
	vertical search window	0.110
Contour preservation	boundary band/pull (η)	5 rings/0.52
	max displacement ($\Delta_{\max}$)	0.048
Local smoothing	boundary iterations/step cap	8–12/0.005

**Table 2:** Mean per-stage runtime (seconds) on the fully crossed 75-case pool at Poisson depth 8. The reported RG-PSR timing covers the core pipeline only; conditional structure-aware repair is excluded. No complete-pipeline runtime is reported.

Method	Reliability	Normals	Poisson Solve	Trim	Total
Poisson + Trim	–	0.29	5.77	0.02	6.08
RG-PSR (core)	0.22	0.43	5.70	0.06	6.43

**Table 3:** Runtime and peak memory as a function of input point count *n* on the bunny mesh at Poisson octree depth 8, measured in an isolated process per configuration. The RG-PSR measurements cover the core pipeline only and exclude conditional structure-aware repair.

Method	n=2k	n=5k	n=10k	n=20k	n=50k
*Runtime* (s)					
Poisson	6.4	7.4	8.2	9.8	14.5
RG-PSR core	7.0	6.7	9.6	11.5	19.1
*Peak RSS* (MB)					
Poisson	641	648	664	695	833
RG-PSR core	642	650	669	697	834

**Table 4:** Precision, recall (completeness/coverage), and F-score at $\tau \;=\;0.01D_{G}$, averaged over the 52 main-benchmark cases. ↑ indicates that higher values are better.

Method	$P_{0.01}$↑	$R_{0.01}$↑	$F_{0.01}$↑
Poisson	0.525	0.816	0.613
Screened Poisson	0.521	0.807	0.607
Poisson + Trim	0.593	0.814	0.662
Alpha Shape	0.679	0.721	0.692
BPA	0.868	0.793	0.825
GP3	0.934	0.772	0.839
RG-PSR	0.633	0.797	0.684

**Table 5:** Poisson-family comparison across three dataset groups. The RG-PSR results correspond to the complete pipeline, including conditional structure-aware repair. Runtime is evaluated separately for the core pipeline in Table 2. RG-PSR improves geometric accuracy and artifact suppression relative to vanilla Poisson and fixed density trimming. Best results are shown in **bold**; ↑ indicates that higher values are better, and ↓ indicates that lower values are better.

Method	Open3D/Stanford		ModelNet10		Procedural CAD		${\mathrm{Artifact}}_{0.02}$ ↓
	Chamfer ↓	$F_{0.01}$ ↑	Chamfer ↓	$F_{0.01}$ ↑	Chamfer ↓	$F_{0.01}$ ↑	
PSR	0.0112	0.7942	0.0652	0.4441	0.0414	0.5382	0.3873
SPSR	0.0113	0.7923	0.0669	0.4359	0.0418	0.5299	0.3894
PSR + Trim	0.0083	0.8245	0.0349	0.5630	0.0272	0.5860	0.3090
**RG-PSR**	**0.0076**	**0.8313**	**0.0331**	**0.6000**	**0.0200**	**0.6138**	**0.2632**

**Table 6:** Focused comparison with classical surface-reconstruction baselines. Chamfer and $F_{0.01}$ summarize point-wise accuracy, while components and largest-component ratio summarize mesh coherence. Best results are shown in **bold**; ↑ indicates that higher values are better, and ↓ indicates that lower values are better.

Method	Overall Chamfer ↓	Overall $F_{0.01}$ ↑	Components ↓	Largest Comp. ↑
Poisson	0.0338	0.6128	25.23	0.9854
Screened Poisson	0.0342	0.6067	24.96	0.9858
Poisson + Trim	0.0218	0.6618	25.27	0.9851
Alpha Shape	0.0084	0.6922	34.40	0.8726
BPA	0.0059	0.8248	85.81	0.6245
GP3	**0.0056**	**0.8390**	176.19	0.6911
RG-PSR	0.0172	0.6836	**16.25**	**0.9872**

**Table 7:** Focused RG-PSR parameter sensitivity on the validation split. All timing values cover the core pipeline only and exclude conditional structure-aware repair. ↑ indicates that higher values are better, and ↓ indicates that lower values are better.

Setting	Value	Chamfer ↓	$F_{0.01}$↑	$F_{0.02}$↑	Core Runtime (s) ↓
trim strength	weak	0.0217	0.6087	0.7491	0.86
trim strength	default	0.0194	0.6288	0.7710	0.87
trim strength	strong	0.0169	0.6572	0.7996	0.90
$\beta_{\max}$	0.4	0.0192	0.6332	0.7757	0.90
$\beta_{\max}$	0.8	0.0193	0.6317	0.7742	0.92
$\beta_{\max}$	1.0	0.0193	0.6319	0.7731	0.91
*k*	16	0.0200	0.6272	0.7668	0.83
*k*	32	0.0193	0.6339	0.7735	0.86
*k*	48	0.0189	0.6342	0.7781	0.96

**Table 8:** RG-PSR core ablation on the fully crossed 75-case pool (15 shapes × 5 degradations) at Poisson depth 8. The conditional structure-aware repair stage is excluded from all rows and all reported runtimes. Best results are shown in **bold**; ↑ indicates that higher values are better, and ↓ indicates that lower values are better.

Method	Chamfer ↓	$F_{0.01}$ ↑	$F_{0.02}$ ↑	${\mathrm{Artifact}}_{0.02}$ ↓	Core Runtime (s) ↓
Poisson	0.0333	0.5812	0.7144	0.3908	5.99
+ filtering	0.0283	0.5943	0.7302	0.3718	6.15
+ normal refinement	0.0327	0.5794	0.7137	0.3901	6.45
+ adaptive trimming	0.0201	0.6436	0.7860	0.2879	6.31
+ filtering + normal refinement	0.0280	0.5928	0.7295	0.3710	6.35
+ normal refinement + trimming	0.0200	0.6415	0.7845	0.2881	6.41
RG-PSR core	**0.0179**	**0.6504**	**0.7946**	**0.2750**	6.43

**Table 9:** Reliability-score validity over 15 shapes. Correlations are within-case Spearman averages; AUROC/AUPRC use $1-r_{i}$ against known synthetic-outlier labels.

Quantity	Value
Within-case ρ($r_{i}$, −point error)	0.025 (positive in 55.6% of cases)
Within-case ρ($r_{i}$, −normal error)	0.462 (positive in 100% of cases)
Outlier detection AUROC	0.940 ± 0.040
Outlier detection AUPRC	0.832 ± 0.088
Mean $r_{i}$ on synthetic outliers	0.133
Mean $r_{i}$ on inlier points	0.547

**Table 10:** LiDAR/scanning application case study evaluated by held-out point-to-mesh distance and coverage. Note that no ground-truth mesh is available; results reflect held-out point coverage rather than absolute surface accuracy. ↑ indicates that higher values are better, and ↓ indicates that lower values are better.

Method	Mean (m) ↓	Cov.@0.05 m ↑	Cov.@0.10 m ↑	Cov.@0.20 m ↑	Artifact ↓
Poisson	0.035	0.786	0.948	0.993	0.213
Poisson + Trim	0.035	0.786	0.948	0.993	0.192
RG-PSR	0.035	0.785	0.946	0.990	0.133

## Data Availability

Degradation scripts and seeds, source code, configurations, and per-case metrics are available from the corresponding author on request. Third-party meshes remain subject to the licenses of the Stanford 3D Scanning Repository, Open3D, and ModelNet10 and are not re-hosted.
